# α2AP regulates vascular alteration by inhibiting VEGF signaling in systemic sclerosis: the roles of α2AP in vascular dysfunction in systemic sclerosis

**DOI:** 10.1186/s13075-017-1227-y

**Published:** 2017-02-03

**Authors:** Yosuke Kanno, En Shu, Hiroyuki Kanoh, Ayaka Matsuda, Mariko Seishima

**Affiliations:** 1 Department of Clinical Pathological Biochemistry, Faculty of Pharmaceutical Science, Doshisha Women’s Collage of Liberal Arts, 97-1 Kodo, Kyo-tanabe, Kyoto, 610-0395 Japan; 2 Department of Dermatology, Gifu University Graduate School of Medicine, 1-1 Yanagido, Gifu, 501-1194 Japan

**Keywords:** Alpha2-antiplasmin, Systemic sclerosis, Vascular dysfunction, VEGF

## Abstract

**Background:**

Systemic sclerosis (SSc) is a connective tissues disease of unknown origin characterized by vascular damage and extensive fibrosis. Recently, we demonstrated that α2-antiplasmin (α2AP) is associated with the development of fibrosis in SSc. We herein investigate the roles of α2AP in vascular dysfunction in SSc.

**Methods:**

Vascular damage in mice was determined by the levels of blood vessels and blood flow. Vascular functions in vascular endothelial cells (ECs) were determined by the levels of tube formation, cell proliferation, and endothelial junction-associated protein (VE-cadherin and PECAM1) production.

**Results:**

The administration of α2AP induced vascular damage in mice. Conversely, the α2AP neutralization improved vascular damage in a bleomycin-induced mouse model of SSc. Additionally, we showed that the SSc fibroblast-conditioned media induced the reduction of tube formation, cell proliferation, and endothelial junction-associated protein production in ECs, and that α2AP neutralization improved them. We also examined the mechanisms underlying the effects of α2AP on vascular alteration in SSc and found that α2AP attenuated vascular endothelial growth factor-induced tube formation, cell proliferation, and endothelial junction-associated protein production through the adipose triglyceride lipase/tyrosine phosphatase SHP2 axis in ECs.

**Conclusion:**

Our findings demonstrate that α2AP is associated with vascular alteration, and that the blocking of α2AP improves vascular dysfunction in SSc.

**Electronic supplementary material:**

The online version of this article (doi:10.1186/s13075-017-1227-y) contains supplementary material, which is available to authorized users.

## Background

Systemic sclerosis (SSc) is a connective tissue disease characterized by vascular damage and fibrosis of skin and visceral organs [1]. Vascular damage, such as the reduction of blood vessels and blood flow, occurs in the early stages of the disease, and leads to extensive fibrosis [2]. However, the detailed mechanisms of SSc pathogenesis is unclear. Vascular endothelial growth factor (VEGF) is known to regulate the growth and activation of vascular endothelial cells (ECs), and plays a critical role in maintaining the vascular function. The expression of VEGF is elevated in various cells, such as fibroblasts, ECs, and immune cells, but vascular insufficiency manifests in SSc [2, 3]. The impairment of VEGF responses may cause vascular dysfunction in SSc. However, the detailed mechanisms are still not precisely understood.

Alpha2-antiplasmin (α2AP) functions as the main inhibitor of plasmin, resulting in the formation of a stable inactive complex, plasmin-α2AP and inhibits fibrinolysis [4]. α2AP is known to be synthesized in various tissues [5]. Recently, we found that α2AP induces TGF-β production through adipose triglyceride lipase (ATGL), which has been described as a member of the calcium-independent phospholipase A_2_/adiponutrin/patatin-like phospholipase domain-containing 2 (PNPLA2) family, and has a pro-fibrotic effects other than regulation of plasmin activity [6–10]. We also found that the expression of α2AP was elevated in the dermal fibroblasts obtained from SSc patients and the fibrotic tissue in SSc mouse models, and α2AP is associated with the development of fibrosis in SSc [7, 10]. Additionally, α2AP is known to play a critical role on angiogenesis, tissue repair, and vascular remodeling [11, 12], and may be also associated with vascular alteration in SSc. We herein investigated that the roles of α2AP in vascular dysfunction in SSc.

## Methods

### Mice experiments

We performed mice experiments as previously described [10]. The saline, bleomycin (5 mg/kg) plus control IgG (100 μg/kg) or bleomycin (5 mg/kg) plus anti-α2AP antibodies (100 μg/kg) (R&D Systems, MN, USA) were administered subcutaneously into the shaved backs of mice (male, 8-week-old C57BL/6 J mice) in the same site daily for up to 3 weeks. In parallel experiments, the saline or α2AP (15 μg/kg) (Calbiochem, CA, USA) were administered subcutaneously into the shaved backs of mice (male, 8-week-old C57BL/6 J mice) in the same site daily for up to 3 weeks. The samples of skin were placed immediately in liquid nitrogen, and stored at −80 °C until further use.

### Immunohistochemical staining of PECAM1

We performed immunohistochemical staining as previously described [10, 11]. Paraffin sections were labeled with anti-PECAM1 antibody, then secondarily labeled with FITC-conjugated anti-rabbit IgG (Thermo Scientific, CA, USA). We used Rabbit (DA1E) mAb IgG XP Isotype control (Cell Signaling Technology, MA, USA) as isotype control (Additional file 1: Figure S1). The signals in the skin section were detected using a laser-scanning microscope. Then, the signals obtained from the same rectangular area for the dermis in the skin section were analyzed using ImageJ.

### Blood flow in the skin

Blood flow in the skin was measured for 10 seconds using a laser Doppler flowmeter (BRL-100; Bio Research Center, Tokyo, Japan), and determined by calculating the average of two-time measurements in each skin sample.

### Cell culture

Human normal and SSc dermal fibroblasts were obtained from patients with SSc (S4) and healthy control (N3) as previously described [10, 11]. Dermal fibroblasts were seeded onto the 10-cm diameter dishes and maintained in 10 mL Dulbecco’s modified Eagle medium (DMEM) containing 10% FCS at 37 °C in a humidified atmosphere with 5% CO_2_/95% air. After 5 days, the media were collected. In other studies, vascular ECs (UV♀2) were seeded onto 35-mm diameter dishes and maintained in 2 mL DMEM containing 10% FCS at 37 °C in a humidified atmosphere with 5% CO_2_/95% air. After 5 days, the media were replaced with serum-free DMEM. Then, the cells were used for experiments.

### Tube formation assay

Matrigel (Becton, Dickinson and Company, NJ, USA) was added to each well of a 96-well plate. ECs were seeded on Matrigel coated plates, and were treated with the conditioned media (CM) of dermal fibroblasts, VEGF, or α2AP at the indicated concentration for 24 hours. The length of capillary like structure was analyzed by using ImageJ.

### Cell proliferation assays

ECs were seeded on a 96-well plate, and the ECs were treated with the CM of dermal fibroblasts, VEGF, or α2AP at the indicated concentration for 24 hours. Cell proliferation was determined by counting cells number.

### Western blot analysis

Cells were washed twice with cold PBS, harvested, and then sonicated in lysis buffer containing 10 mM Tris–HCl buffer (pH 7.5), 1% SDS, 1% Triton X-100, and a protease inhibitor cocktail (Roche, Mannheim, Germany). The skin samples from mice were homogenized and sonicated in the lysis buffer. The protein concentration in each lysate was measured using a BCA protein assay kit (Pierce, IL, USA). Proteins in the supernatant were separated by electrophoresis on 10% SDS-polyacrylamide gels and transferred to a PVDF membrane. We detected PECAM1, vascular endothelial cadherin, GAPDH, phospho-VEGFR2, VEGFR2, phospho-Akt, Akt, phospho-ERK1/2, ERK1/2, phospho-p38, p38, phospho-SHP2, SHP2, and ATGL by incubation with the respective antibodies followed by incubation with horseradish peroxidase-conjugated antibodies to rabbit IgG (Amersham Pharmacia Biotech, Uppsala, Sweden).

### ATGL siRNAs study

SSc dermal fibroblasts were transfected with ATGL siRNA (Santa Cruz Biotechnology, CA, USA) using Lipofectamine 2000 (Invitrogen, CA, USA) according to the manufacturer’s instructions. A non-specific siRNA was employed as the control. At 24 hours after transfection, the cells were used for experiments.

### Statistical analysis

All data were expressed as mean ± SEM. The significance of the effect of each treatment (*P* < 0.05) was determined by analysis of variance (ANOVA) followed by the least significant difference test.

## Results

### Effect of α2AP on vascular damage in mice

**Fig. 1:**
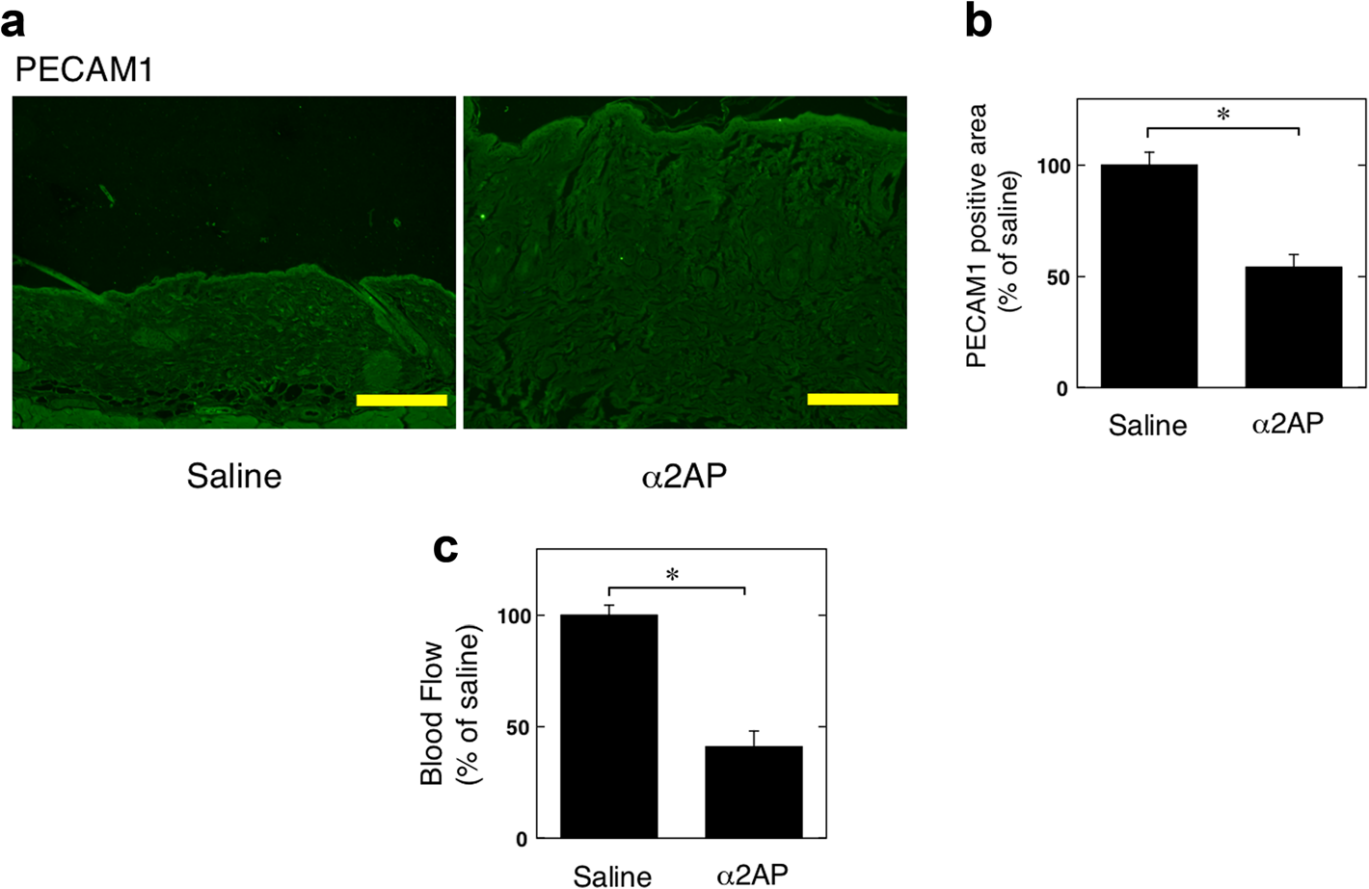
Effect of α2AP on vascular damage in mice. **a** The skin sections from saline or α2AP-administered mice were stained with antibodies to PECAM1. **b** The histogram shows quantitative representations of PECAM1 (n = 6). **c** Blood flow in the skin of saline or α2AP-administered mice (n = 4). The data represent the mean ± SEM. * *P* < 0.01. Scale bar, 200 μm

To clarify the effects of α2AP on vascular damage, such as the reduction of blood vessels and blood flow, we examined the expression of the vascular EC marker, platelet-endothelial cell adhesion molecule 1 (PECAM1, CD31) and blood flow following the administration of α2AP in mice. The administration of α2AP induced the reduction of PECAM1 expression within the dermis (Fig. 1a, b) and blood flow in the skin (Fig. 1c).

### Effect of blocking α2AP on vascular damage in a bleomycin-induced mouse model of SSc

**Fig. 2:**
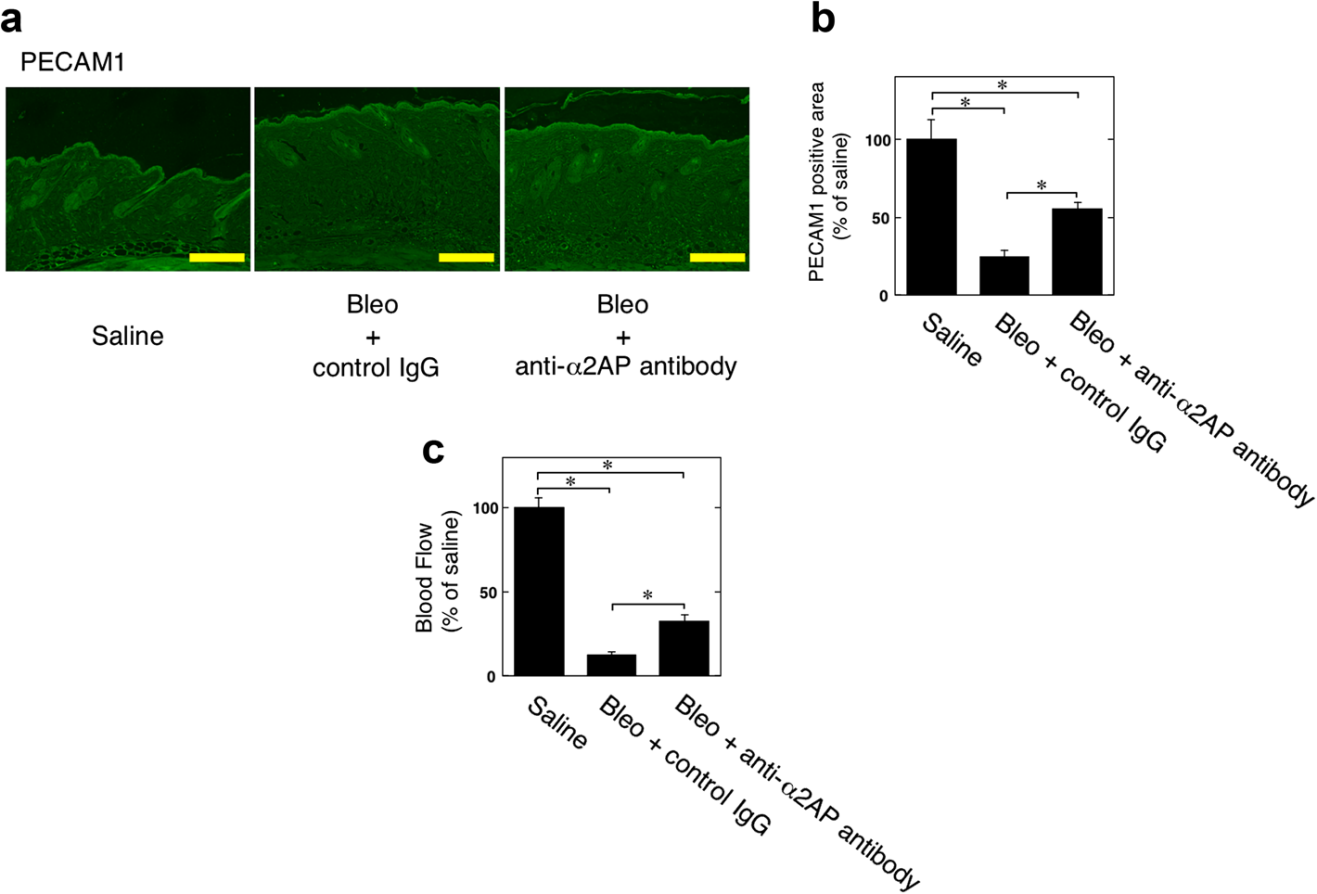
Effect of blocking α2AP on vascular damage in a bleomycin-induced mouse model of systemic sclerosis. **a** The skin sections from mice treated with saline, bleomycin plus control IgG, or bleomycin plus α2AP-neutralizing antibodies were stained with antibodies to PECAM1. **b** The histogram shows quantitative representations of PECAM1 (n = 6). **c** Blood flow in the skin of mice treated with saline (n = 5), bleomycin plus control IgG (n = 4), or bleomycin plus α2AP-neutralizing antibodies (n = 4). The data represent the mean ± SEM. * *P* < 0.01. Scale bar, 200 μm

We examined the effects of α2AP neutralization on vascular damage in a bleomycin-induced mouse model of SSc. The administration of bleomycin induced the reduction of PECAM1 expression (Fig. 2a, b) and blood flow (Fig. 2c) in the skin of SSc model mice, and the blocking of α2AP by α2AP-neutralizing antibodies improved the bleomycin-induced reduction of PECAM1 expression within the dermis (Fig. 2a, b) and blood flow (Fig. 2c) in the skin of SSc model mice.

### Effect of blocking α2AP on the SSc dermal fibroblast-induced vascular dysfunction in ECs

**Fig. 3:**
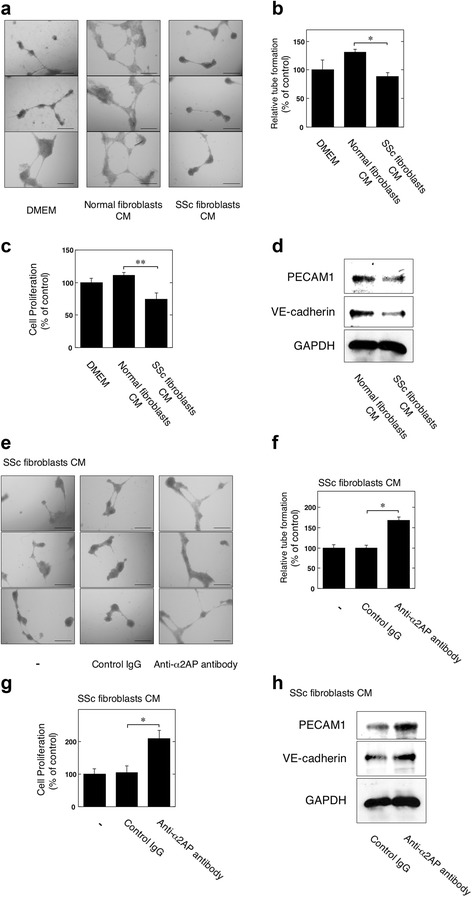
Effect of blocking α2AP on the systemic sclerosis (SSc) dermal fibroblast-induced vascular dysfunction in endothelial cells (ECs). **a** ECs were seeded on 96-well Matrigel coated plates. ECs were cultured with the condition media (CM) of human normal dermal fibroblasts or human SSc dermal fibroblasts for 24 hours. **b** Tube formation in ECs was measured as described in Materials and Methods (n = 6). **c** ECs were cultured with the CM of the human normal dermal fibroblasts or the human SSc dermal fibroblasts for 24 hours. The cell proliferation was assessed as described in Materials and Methods (n = 3). **d** ECs were cultured with the CM of human normal dermal fibroblasts or human SSc dermal fibroblasts for 24 hours. The expression of each protein was examined by western blot analysis. **e** ECs were seeded on 96-well Matrigel-coated plates. ECs were cultured with the CM of human SSc dermal fibroblasts, and were stimulated by control IgG (1 μg/mL) or α2AP-neutralizing antibodies (1 μg/mL) for 24 hours. **f** Tube formation in ECs was measured as described in Materials and Methods (n = 6). **g** ECs were cultured with the CM of the human SSc dermal fibroblasts, and were stimulated by control IgG (1 μg/mL) or α2AP-neutralizing antibodies (1 μg/mL) for 24 hours. Cell proliferation was assessed as described in Materials and Methods (n = 8). **h** ECs were cultured with the CM of human SSc dermal fibroblasts and were stimulated by control IgG (1 μg/mL) or α2AP-neutralizing antibodies (1 μg/mL) for 24 hours. The expression of each protein was examined by western blot analysis. Data represent the mean ± SEM. * *P* < 0.01, ** *P* < 0.05. Scale bar, 50 μm

We examined whether or not the CM from SSc dermal fibroblasts induces vascular dysfunction, including the reduction of tube formation, cell proliferation, and endothelial junction-associated protein (PECAM1 and vascular endothelial cadherin) production. The tube formation (Fig. 3a, b), cell proliferation (Fig. 3c), and endothelial junction-associated protein production (Fig. 3d) in SSc dermal fibroblast CM-stimulated ECs were lower than those in normal dermal fibroblast CM-stimulated ECs. These data suggest the SSc dermal fibroblasts induced vascular dysfunction. Next, we examined the effects of α2AP neutralization on the SSc dermal fibroblast CM-induced reduction of tube formation, cell proliferation, and endothelial junction-associated protein production. The α2AP neutralization improved the SSc dermal fibroblast CM-induced reduction of tube formation (Fig. 3e, f), cell proliferation (Fig. 3g), and endothelial junction-associated protein production (Fig. 3h).

### Effect of α2AP on the VEGF-induced pro-angiogenic effects in ECs

**Fig. 4:**
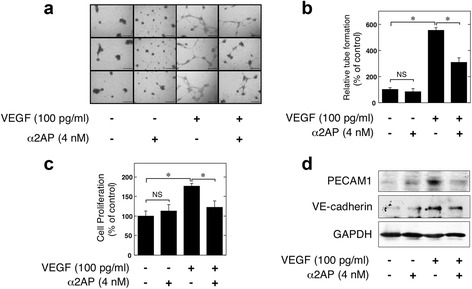
Effect of α2AP on the VEGF-induced pro-angiogenic effects in endothelial cells (ECs). **a** ECs were seeded on 96-well Matrigel-coated plates. ECs were cultured in the absence or presence of VEGF (100 pg/mL) or α2AP (4 nM) as indicated for 24 hours. **b** Tube formation in ECs was measured as described in Materials and Methods (n = 6). **c** ECs were cultured for 24 hours in the absence or presence of VEGF (100 pg/mL) or α2AP (4 nM). Cell proliferation was assessed as described in Materials and Methods (n = 3). **d** ECs were cultured for 24 hours in the absence or presence of VEGF (100 pg/mL) or α2AP (4 nM). The expression of each protein was examined by western blot analysis. The data represent the mean ± SEM. * *P* < 0.01. NS, not significant. Scale bar, 50 μm

We examined the effects of α2AP on the VEGF-regulated tube formation, cell proliferation, and endothelial junction-associated protein production in ECs. We confirmed that VEGF induced tube formation (Fig. 4a, b), cell proliferation (Fig. 4c), and endothelial junction-associated protein production (Fig. 4d) in ECs, and found that α2AP attenuated VEGF-induced tube formation (Fig. 4a, b), cell proliferation (Fig. 4c), and endothelial junction-associated protein production (Fig. 4d) in ECs.

### Effect of α2AP on VEGF signaling through ATGL/SHP2 axis in ECs

**Fig. 5:**
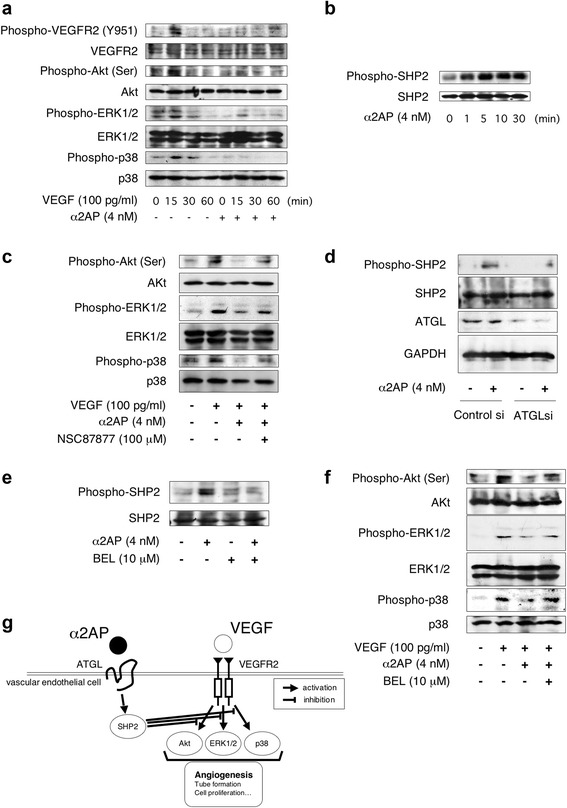
Effect of α2AP on the VEGF signaling through ATGL/SHP2 axis in endothelial cells (ECs). **a** ECs were pretreated with 4 nM α2AP for 30 minutes and then stimulated with 100 pg/mL VEGF for the indicated periods. Phosphorylation of each protein was examined by western blot analysis. **b** ECs were stimulated with 4 nM α2AP for the indicated periods. Phosphorylation of SHP2 was examined by western blot analysis. **c** ECs were cultured for 30 minutes in the absence or presence of 4 nM α2AP or 100 μM NSC87877, and then stimulated with 100 pg/mL VEGF for 15 minutes. Phosphorylation of each protein was examined by western blot analysis. **d** ECs were transfected with control or ATGL siRNA. At 24 hours after transfection, the cells were stimulated with 4 nM α2AP for 5 minutes. The expression of each protein was examined by western blot analysis. **e** ECs were pretreated with 10 μM BEL for 30 minutes and then stimulated with 4 nM α2AP for 5 minutes. Phosphorylation of SHP2 was examined by western blot analysis. **f** ECs were pretreated with 4 nM α2AP or 10 μM BEL for 30 minutes and then stimulated with 100 pg/mL VEGF for 15 minutes. Phosphorylation of each protein was examined by western blot analysis. **g** The proposed mechanism of α2AP-attenuated vascular endothelial functions. VEGF induced Akt, ERK1/2, and p38 activation and led to pro-angiogenic effects, such as tube formation, cell proliferation, and endothelial junction-associated protein production in ECs. On the other hand, α2AP activated SHP2 through ATGL, and then α2AP-induced SHP2 activation inhibited VEGF signaling in ECs. α2AP inhibited VEGF signaling through the ATGL/SHP2 axis, and may cause impairment of vascular functions

We also examined the effects of α2AP on VEGF signaling in ECs, and found that α2AP inhibited VEGF-induced VEGFR2, Akt, ERK1/2, and p38 phosphorylation in ECs (Fig. 5a). It has been reported that src-homology domain-2 containing tyrosine phosphatase 2 (SHP2) activation inhibits VEGF signaling [13]. Therefore, we examined whether or not α2AP-inhibited VEGF signaling is associated with SHP2 activation in ECs. α2AP induced SHP2 phosphorylation (Fig. 5b) and the SHP2 inhibitor, NSC87877, abrogated α2AP-inhibited Akt, ERK1/2, and p38 phosphorylation induced by VEGF (Fig. 5c). We then examined whether or not the α2AP-induced SHP2 activation is associated with the α2AP receptor, ATGL, in ECs. The reduction of ATGL using siRNA attenuated the α2AP-induced SHP2 phosphorylation in ECs (Fig. 5d). We also examined the effects of the irreversible ATGL inhibitor, bromoenol lactone (BEL), on α2AP-activated SHP2 in ECs. BEL attenuated α2AP-induced SHP2 phosphorylation in ECs (Fig. 5e). Additionally, BEL abrogated α2AP-inhibited Akt, ERK1/2, and p38 phosphorylation induced by VEGF in ECs (Fig. 5f).

## Discussion

SSc is a chronic immune disorder characterized by vascular dysfunction and fibrosis of the skin and internal organs [1]. Recently, we showed that α2AP is associated with the development of fibrosis in SSc [6–8, 10]. α2AP is also associated with angiogenesis [11], vascular remodeling [12], the production of IgG, IgM, and IgE [14, 15], and the recruitment of lymphocytes and neutrophils [15–17]. These observations suggest that α2AP may be a critical regulator in the pathogenesis of SSc. We herein demonstrated that α2AP is associated with vascular dysfunction in SSc.

We showed that the administration of α2AP induced vascular damage such as the reduction of blood vessels and blood flow in mice (Fig. 1). Conversely, α2AP neutralization improved vascular damage in a bleomycin-induced mouse model of SSc (Fig. 2). These data suggest that α2AP may be one of the factors initiating vascular damage in SSc.

In SSc, fibroblasts are likely to be important effector cells, and SSc fibroblasts inhibit angiogenesis [18, 19]. We therefore examined whether or not SSc fibroblasts induce vascular dysfunction, such as the reduction of tube formation, cell proliferation, and endothelial junction-associated protein production, using CM from human normal and SSc dermal fibroblasts. We found that SSc dermal fibroblasts induced vascular dysfunction (Fig. 3a–d). We also showed that the blocking of α2AP markedly improved SSc dermal fibroblast-induced vascular dysfunction (Fig. 3e–h). In a previous study, we showed that the expression of α2AP was elevated in SSc dermal fibroblasts [10]. The SSc fibroblast-derived α2AP may cause vascular dysfunction in the disease.

It has been reported that the expression of VEGF, which is a main regulator of angiogenesis, is elevated in SSc patients [2, 3]. However, angiogenesis is disturbed in SSc, and the mechanism of dysregulated angiogenesis in the presence of elevated VEGF remains poorly understood. We showed that α2AP attenuated VEGF-induced pro-angiogenic effects such as tube formation, cell proliferation, and endothelial junction-associated protein production in ECs (Fig. 4). Additionally, we showed that α2AP inhibited VEGF signaling (VEGFR2, Akt, ERK1/2, and p38 activation) (Fig. 5a). It has been reported that the activation of SHP2 inhibits VEGF signaling and regulates vascular endothelial functions [13]. In this study, we found that α2AP induced SHP2 activation (Fig. 5b), and the inhibition of SHP2 recovered α2AP-attenuated VEGF signaling (Fig. 5c). We also found that α2AP inhibited VEGF signaling through SHP2 activation. We previously showed that α2AP induces cell differentiation and TGF-β production through ATGL [8]. Therefore, we examined whether or not ATGL is associated with α2AP-induced SHP2 activation using siRNA and its inhibitor. Both reduction and inhibition of ATGL attenuated the α2AP-induced SHP2 activation (Fig. 5d, e). Additionally, the inhibition of ATGL recovered the α2AP-inhibited VEGF signaling (Fig. 5f). These data suggest that α2AP induced SHP2 activation through ATGL, and the α2AP-activated SHP2 inhibited VEGF signaling (Fig. 5g). The increase of α2AP expression in SSc may cause impairment of the VEGF response, and lead to vascular dysfunction.

Additionally, plasmin is known to regulate vascular endothelial functions, and influence the progression of various cardiovascular diseases through fibrinolysis, the degradation of matrix proteins, and the activation of growth factors [20]. The levels of plasmin-α2AP and D-dimer are elevated in patients with SSc [21, 22], and plasmin may also affect vascular dysfunction in SSc. α2AP may cause vascular disorder not only through inhibition of VEGF responses but also through plasmin inhibition.

## Conclusion

α2AP functions as an inducer of vascular damage in mice. Blocking of α2AP improved vascular damage in an SSc mice model and SSc dermal fibroblast-induced vascular dysfunction. Additionally, α2AP regulated vascular alteration by inhibiting VEGF signaling through the ATGL/SHP2 axis. Our findings may eventually provide new insights into the development of clinical therapies for SSc.

## Additional file

Additional file 1: Figure S1. (A) The skin sections from saline or α2AP-administered mice were stained with isotype control. (B) The skin sections from mice treated with saline, bleomycin plus control IgG, or bleomycin plus α2AP-neutralizing antibodies were stained with isotype control. Scale bar, 200 μm. (TIF 1805 kb)

## Abbreviations

ATGL: adipose triglyceride lipase
BEL: bromoenol lactone
CM: conditioned media
EC: endothelial cell
PECAM1: platelet-endothelial cell adhesion molecule 1
PNPLA2: patatin-like phospholipase domain-contain 2
SHP2: src-homology domain-2 containing tyrosine phosphatase 2
SSc: systemic sclerosis
VEGF: vascular endothelial growth factor
α2AP: α2-antiplasmin
