# Supplementary figures and images for: α2AP regulates vascular alteration by inhibiting VEGF signaling in systemic sclerosis: the roles of α2AP in vascular dysfunction in systemic sclerosis
Source: Arthritis Res Ther. 2017 Feb 3;19:22. doi: 10.1186/s13075-017-1227-y (PMC5291960; doi:10.1186/s13075-017-1227-y)

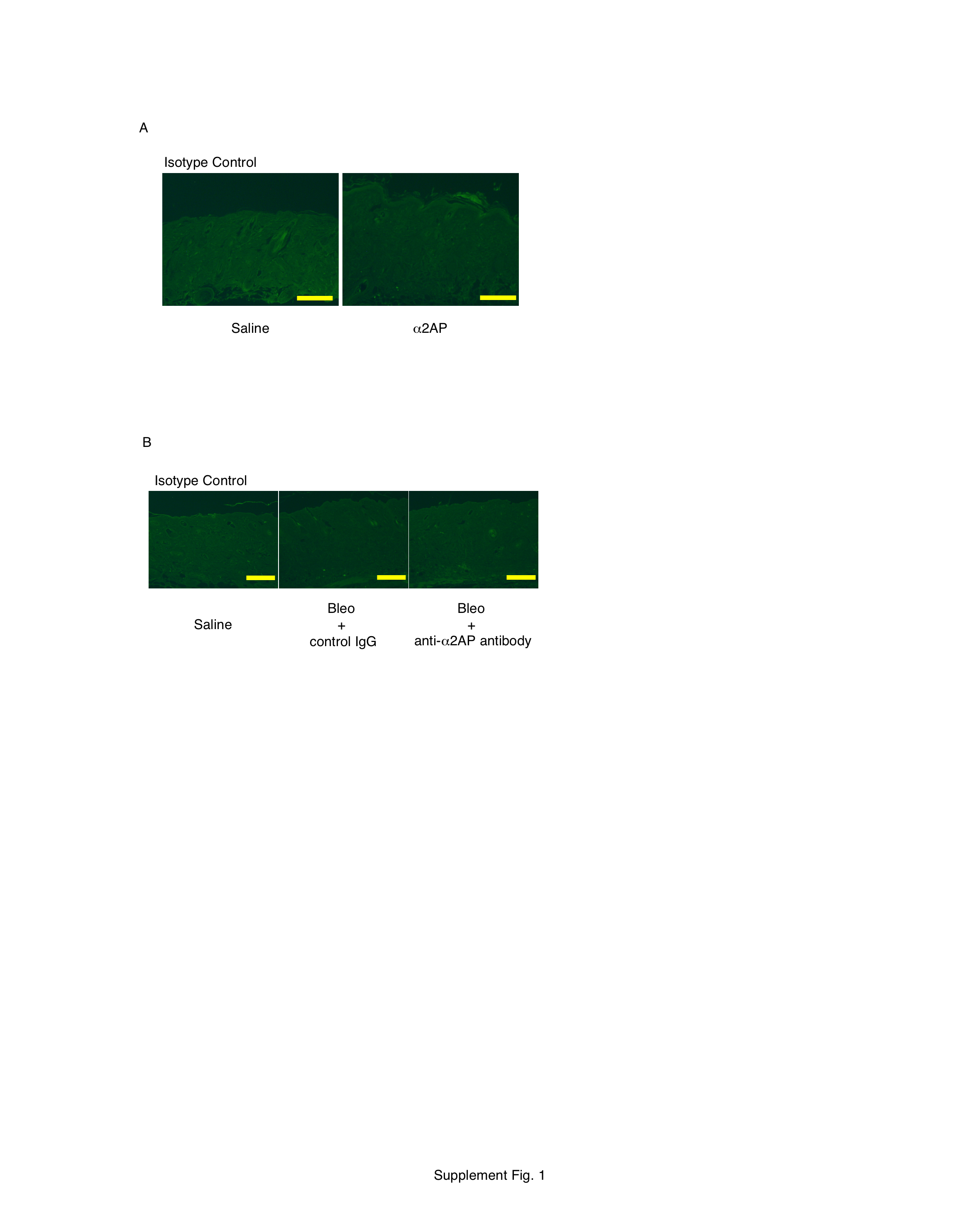

Supplement: Additional file 1: Figure S1. — (A) The skin sections from saline or α2AP-administered mice were stained with isotype control. (B) The skin sections from mice treated with saline, bleomycin plus control IgG, or bleomycin plus α2AP-neutralizing antibodies were stained with isotype control. Scale bar, 200 μm. (TIF 1805 kb) [file 13075_2017_1227_MOESM1_ESM.tif]
